# MicroRNA-146a gene transfer ameliorates senescence and senescence-associated secretory phenotypes in tendinopathic tenocytes

**DOI:** 10.18632/aging.205505

**Published:** 2024-02-02

**Authors:** Che-Chia Hsu, Shih-Yao Chen, Po-Yen Ko, Fa-Chuan Kwan, Wei-Ren Su, I-Ming Jou, Po-Ting Wu

**Affiliations:** 1 Department of Orthopaedics, National Cheng Kung University Hospital, College of Medicine, National Cheng Kung University, Tainan, Taiwan; 2 Department of Nursing, College of Nursing, Chung Hwa University of Medical Technology, Tainan, Taiwan; 3 Department of Orthopaedics, E-Da Hospital, Kaohsiung, Taiwan; 4 School of Medicine, College of Medicine, I-Shou University, Kaohsiung, Taiwan; 5 GEG Orthopedic Clinic, Tainan, Taiwan; 6 Medical Device Innovation Center, National Cheng Kung University, Tainan, Taiwan; 7 Department of Orthopaedics, College of Medicine, National Cheng Kung University, Tainan, Taiwan; 8 Department of Biomedical Engineering, National Cheng Kung University, Tainan, Taiwan; 9 Department of Biochemistry and Molecular Biology, College of Medicine, National Cheng Kung University, Tainan, Taiwan

**Keywords:** tendinopathy, senescence, senescence-associated secretory phenotypes, microRNA-146a, lentiviral vector

## Abstract

Objective: Tendinopathy is influenced by multiple factors, including chronic inflammation and aging. Senescent cells exhibit characteristics such as the secretion of matrix-degrading enzymes and pro-inflammatory cytokines, collectively known as senescence-associated secretory phenotypes (SASPs). Many of these SASP cytokines and enzymes are implicated in the pathogenesis of tendinopathy. MicroRNA-146a (miR-146a) blocks senescence by targeting interleukin-1β (IL-1β) receptor–associated kinase 4 (IRAK-4) and TNF receptor–associated factor 6 (TRAF6), thus inhibiting NF-κB activity. The aims of this study were to (1) investigate miR-146a expression in tendinopathic tendons and (2) evaluate the role of miR-146a in countering senescence and SASPs in tendinopathic tenocytes.

Methods: MiR-146a expression was assessed in human long head biceps (LHB) and rat tendinopathic tendons by *in situ* hybridization. MiR-146a over-expression in rat primary tendinopathic tenocytes was achieved by lentiviral vector-mediated precursor miR-146a transfer (LVmiR-146a). Expression of various senescence-related markers was analyzed by quantitative reverse transcription polymerase chain reaction (qRT-PCR), immunoblotting and immunofluorescence. MiR-146a expression showed a negative correlation with the severity of tendinopathy in human and rat tendinopathic tendons (p<0.001).

Results: Tendinopathic tenocyte transfectants overexpressing miR-146a exhibited downregulation of various senescence and SASP markers, as well as the target molecules IRAK-4 and TRAF6, and the inflammatory mediator phospho-NF-κB. Additionally, these cells showed enhanced nuclear staining of high mobility group box 1 (HMGB1) compared to LVmiR-scramble-transduced controls in response to IL-1β stimulation.

Conclusions: We demonstrate that miR-146a expression is negatively correlated with the progression of tendinopathy. Moreover, its overexpression protects tendinopathic tenocytes from SASPs and senescence through the IRAK-4/TRAF6/NF-kB pathway.

## INTRODUCTION

Tendinopathy is a clinical condition characterized by pain and significant dysfunction in tendons. Despite extensive research, the precise pathogenic mechanism of tendinopathy remains unclear. It is believed to result from a combination of various factors, including overuse [1], chronic inflammation [2], apoptosis [3], and aging [4]. Cellular senescence, a hallmark of aging, has emerged for dissecting the pathogenic mechanisms in osteoarthritis (OA) and tendinopathy. Senescence-associated secretory phenotypes (SASPs), characterized by increased expression of proinflammatory mediators such as IL-6 [5], and matrix-degrading enzymes, including matrix metalloproteinase-1 (MMP)-1 and -3 [6], play a crucial role in OA [7–9]. Targeting senescence has been proposed as an ideal therapeutic approach for tendinopathy [10–12]. Anti-senescent therapies for tendinopathy encompass various synergistic effects, including anti-inflammation [10], anti-apoptosis [12], suppressing oxidative stress [13], maintaining extracellular matrix homeostasis [11], and promotion of the sufficient differentiation of tendon stem/progenitor cells into tenocytes [14].

MicroRNAs (miRNAs) are small, non-protein coding single-stranded RNA molecules, which function as posttranslational regulators of the specific messenger RNAs (mRNAs). They promote mRNA degradation through perfect complementarity or translational repression through partial complementarity with the 3'-untranslated region (UTR) [15]. One such miRNA, miR-146a, was the first identified differentially expressed miRNAs in OA. MiR-146a-deficent mice developed early onset of OA characteristics whereas chondrogenic over-expression of miR-146a made mice resistant to aging-related and post-traumatic OA [16]. Interleukin-1β (IL)-1β is known to mediate catabolism in OA articular cartilage and tendon-derived cells by increasing levels of matrix-degrading enzymes and pro-inflammatory cytokines through NF-κB activation [17–19]. MiR-146a blocked activation of the aforementioned pathway by targeting IL-1 receptor–associated kinase 1 (IRAK-1), IRAK-4 and TNF receptor–associated factor 6 (TRAF6), thus resolving inflammation by blocking NF-κB activity [20, 21]. In a previous study, we demonstrated the protective role of CD44, the major receptor of hyaluronate (HA), in tenocytes by inhibiting NF-κB activation and SASPs induced by IL-1β [11]. Furthermore, miR-146a was reported to be induced by HA engagement in OA chondrocytes [22]. Based on these findings, we hypothesize that miR-146a is involved in the pathogenesis of tendinopathy and may alleviate senescence in tendinopathic tenocytes through the IRAK4/TRAF6/NF-kB pathway. The aims of this study are to (i) investigate miR-146a expression in tendinopathic tendons and (ii) evaluate the role of miR-146a in countering senescence and SASPs in tendinopathic tenocytes.

## MATERIALS AND METHODS

### Collection of clinical specimens

Ten consecutive patients (3 men, 7 women; mean age, 66.10 ± 2.15 years; median age, 69; age range, 52–74 years) undergoing arthroscopic treatment for LHB tendinopathy at our university hospital were recruited. Human hamstring tendons were collected from 10 consecutive patients (8 men, 2 women; mean age: 40 ± 3.58 years; median age, 38; age range: 27–64 years) undergoing anterior cruciate ligament reconstruction using autologous hamstring tendons as controls.

### Tendinopathic tendon and tenocyte from collagenase-injected rat Achilles tendon

For collection of tendinopathic tendon samples, 8-week-old male Sprague-Dawley rats (weighting 250–300 g) were purchased from LASCO, Taiwan, and housed in the Laboratory Animal Center, College of Medicine, National Cheng Kung University, and Taiwan Animal Consortium (AAALAC International Full Accreditation). The healthy conditions of the rats were monitored strictly by administrators at our center. Rats were intratendinously injected with 10 μL (0.015 mg/μL in 0.9% saline) bacterial collagenase I (Sigma-Aldrich, St. Louis, MO, USA) into their right Achilles tendons using a 29G needle to induce tendinopathy, as described previously [11, 23]. Eight weeks after the index procedure, the model was considered mature, and the injected Achilles tendons were harvested for further analysis after euthanizing the rats with an overdose of isoflurane. For primary culture of tendinopathic tenocytes, male Sprague–Dawley rats (4 – 6 weeks old) were adopted and received intratendinous injections of collagenase I (0.015 mg/μL, 10 μL injection/rat, Sigma-Aldrich). One week after the index procedure, Achilles tendons were harvested after euthanizing the rats. The preparation of tendon samples and tenocyte culture methods were consistent with those in our previous studies [3, 11].

### Histopathological, immunofluorescence, and *in situ* hybridization (ISH) analyses

The human and rat specimens were fixed in fresh 4% paraformaldehyde for 16–24 h at 4° C, dehydrated, paraffin-embedded, and longitudinally sectioned. Sequential 4-μM sections were stained with hematoxylin and eosin (H&E) and examined under a light microscope to assess changes in tenocyte morphology and collagen-bundle characteristics. A simple semiquantitative scoring system based on tenocyte morphology and collagen-bundle characteristics and using a 4-point scale (0–3), was employed to classify tendinopathy into normal, mild, moderate, and severe grades (0, ≤2, 3–4, and ≥5 points) [3, 11]. Histological grading was assessed by three observers who were blinded to the clinical or experimental settings. In cases of inconsistency, the field was reassessed, and a final score was determined. For immunofluorescence staining, the tenocytes with various treatments were fixed using 4% paraformaldehyde, permeabilized with 0.5% Triton X-100, blocked with 5% bovine serum albumin, and then stained with an antibody against HMGB1 (Santa Cruz Biotechnology Dallas, TX, USA), followed by the Alexa Flour 488-conjugated secondary antibody (Thermo Fisher Scientific, Waltham, MA, USA) and observed under a fluorescence microscope. For the ISH procedure, the sections were first deparaffinized, followed by fixation in paraformaldehyde. They were then treated with proteinase K, acetylated with acetic anhydride in triethanolamine hydrochloride, and washed in PBS. Next, the sections were incubated in prehybridization buffer (BioChain, Newark, CA, USA) and hybridized with LNA digoxigenin (DIG)-labeled probes (Exiqon, Woburn, MA, USA) for miR-146a and U6 (used as an internal control). An alkaline phosphatase-conjugated anti-DIG antibody was applied, and the sections were stained with 5-Bromo-4-chloro-3-indolyl phosphate (BCIP)/nitroblue tetrazolium (NBT) [24]. The percentage of miR-146a-positive cells in LHB specimens was calculated by counting the number of purple-colored containing cells relative to the number of pink-colored total cells. In each patient, the percentage of miR-146a-positive cell was recorded in three randomly selected high-power field (x400) corresponding to each histopathological severity. In some cases, only one or two disease severities are presented in the specimens. Furthermore, two patients who presented no miR-146a-positive cell in any histopathological severity field were excluded from the analysis of *in situ* hybridization of miR-146a. Finally, the mean value and standard error for each severity grade was determined based on the data collected from all patients (Supplementary Table 1).

### Generation of lentiviral vectors that stably expressing miR-146a

The lentiviral plasmid that expressed precursor miR-146a (NCBI GenBank ID: 100314241) was purchased from System Bioscience (SBI) (Palo Alto, CA, USA). Recombinant lentiviruses LVmiR-146a were produced by transient transfection of 293T cells with PMIRH146a PA 1 2, along with the packaging plasmid psPAX2 and the envelope plasmid pMD2.G encoding vesicular stomatitis virus G glycoprotein (VSV-G) using calcium phosphate precipitation methods as previously described [24, 25]. After 48 h, lentiviral particles were collected and concentrated from the supernatants by ultracentrifugation. Physical titers of lentiviruses, expressed as viral particles (VP), were determined by the analysis of virus-associated p24 core protein concentrations with the QuickTiter Lentivirus titer kit (Cell Biolabs, San Diego, CA, USA) [24, 25].

### Generation of rat tenocytes stably expressing miR-146a and stimulation

To create miR-146a-overexpressing cells, rat tendinopathic tenocytes were infected with lentiviruses encoding the precursor miR-146a or scramble sequences (used as a control virus) for 72 h, respectively. MiR-146a expression was examined by qRT-PCR with a TaqMan MicroRNA Assay kit and TaqMan Universal PCR Master Mix (Applied Biosystems, Waltham, MA, USA). The expression levels of miR-146a were analyzed using U6 small RNA as an internal control and the 2^-∆∆CT^ method was employed [24, 25]. MiR-146a and scramble control-expressed rat tenocytes were stimulated with IL-1β (10 ng/ml) for 96h and the downstream signaling molecules were examined by qRT-PCR or immunoblot analysis.

### QRT-PCR and immunoblot analyses

Total RNA from rat tenocytes was isolated using TRIzol reagents (#15596018, Invitrogen, USA), and complementary DNA was synthesized using the High Capacity cDNA Reverse Transcription Kit (#4368813, Applied Biosystems) for qRT-PCR with the SYBR Green PCR kit (#208054, Qiagen, The Netherlands) and specific primer pairs for IL-6 (forward, 5’-CGAAAGTCAACTCCATCTGCC-3’; reverse, 5’-G GCAACTGGCTGGAAGTCTCT-3’), COX-2 (forward, 5′-CACGGACTTGCTCACTTTGTT-3′; reverse, 5′-AAGCGTTTGCGGTACTCATT-3′) and glyceraldehyde-3-phosphate dehydrogenase (GAPDH) (forward, 5’-CCATCTTCCAGGAGCGAGATC-3’; reverse, 5’-GCCTTCTCCATGGTGGTGAA-3’). The comparative Ct method was used to calculate the relative abundance of each gene compared with GAPDH expression [3, 11]. Cell lysates of tenocytes receiving various treatments (IL-1β stimulation, LVmiR-scramble, and LVmiR-146a infection) were subjected to immunoblot analysis using antibodies against TRAF6 (1:1000, Santa Cruz), IRAK-4 (1:1000, Santa Cruz), p16 (1:1000, Santa Cruz), p21 (1:1000, Santa Cruz), p53 (1:1000, Cell Signaling Technology, USA), phospho-NF-ĸB (1:1000, Cell Signaling Technology), NF-ĸB (1:1000, Santa Cruz), MMP-1 (1:1000, GeneTex, USA), and -3 (1:1000, Cell Signaling Technology), in combination with horseradish-peroxidase conjugated secondary antibodies (1:10000, Jackson ImmunoResearch, USA) and quantitative control anti-β-actin antibodies (Sigma-Aldrich). Protein-protein complexes were visualized using an ECL Plus System (Amersham, UK) and analyzed using a BioSpectrum Imaging System (UVP) for chemiluminescence detection. The signal intensity was further quantified by densitometry, and the relative abundance of each gene was compared with β-actin expression [3, 11].

### Senescence assay

Tendinopathic tenocytes were infected with LVmiR-scramble and LVmiR-146a for 72 h. Tenocyte transfectants in which miR-146a were overexpressed, were stimulated with IL-1β (10 ng/mL, R&D Systems, Minneapolis, MN, USA) for 5 days, and their senescent statuses were evaluated using the Senescence β-Galactosidase Staining Kit (#9860, Cell Signaling Technology). SA-β-gal was identified and counted in five high-power fields (200×) to determine the average percentages of SA-β-gal-positive cells corresponding to the total number of cells [11].

### Statistical analysis

Data are expressed as the mean ± SEM. Normality was passed in each data point using the Shapiro-Wilk test. Differences among groups were analyzed using one-way analysis of variance, followed by Dunnett’s multiple comparison test (Prism 5.0). The significance of the correlation between the ratios of miR-146a-positive cells and the histopathological grades of tendinopathy was assessed using the Spearman correlation rank test. p < 0.05 was statistically significant.

### Data availability statement

All analyzed data were showed in the main manuscript. The raw data generated during the current study are available from the corresponding author on reasonable request.

## RESULTS

### MiR-146a expression in human tendons

**Figure 1:**
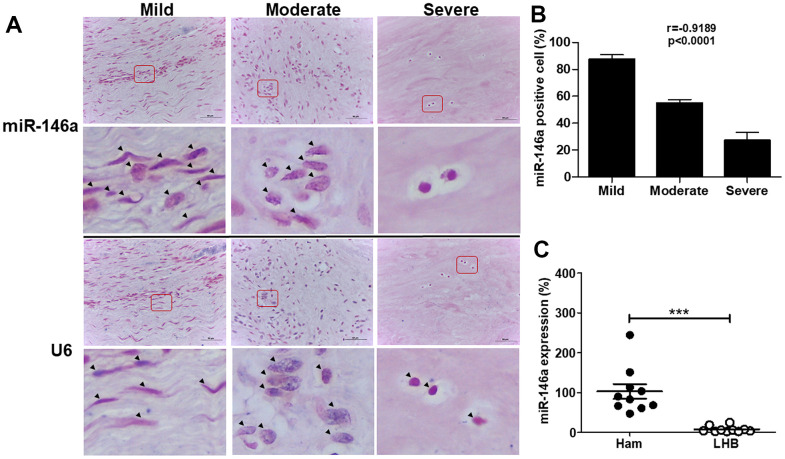
**miR-146a expression in tendon tissues from human long head of biceps (LHB) with tendinopathic changes.** (**A**) Representative figures of *in situ* hybridization of miR-146a in LHB tendons with different severity grades. Arrow heads indicate miR-146a- or U6-positive cells. Scale bars represent 50 μm in × 400 magnifications. The higher-magnification views of the red-boxed areas are shown beneath them. (**B**) miR-146a-positive cells were counted and normalized with U6-counterstained total cells. Spearman correlation rank test was used. (**C**) miR-146a expression levels in tendon tissues of normal hamstring (Ham) tendon during anterior cruciate ligament reconstruction and LHB, as determined by quantitative reverse-transcription polymerase chain reaction (qRT-PCR). Values are represented as the mean ± standard error of the mean (SEM). n=10 each, ***p<0.001.

To examine the role of miR-146a in tendinopathy progression, we assessed miR-146a expression in tendinopathic long head biceps (LHB) tendons by *in situ* hybridization (Figure 1A) and quantitative reverse transcription polymerase chain reaction (qRT-PCR) (Figure 1C). The histological sections of LHB tendons were graded from mild to severe based on a semiquantitative scoring system [3, 11]. MiR-146a exhibited strong staining in tenocyte-like cells in the mild grade sections but was rarely observed in chondrocyte-like cells in the severe grade sections (Figure 1A). Quantitative analysis demonstrated a negative correlation between miR-146a expression levels and the histological grades of tendinopathy, indicating reduced miR-146a expression with disease progression (r = -0.8563, p<0.001, Figure 1B). Additionally, we observed lower miR-146a expression in tendinopathic LHB tendons compared to normal hamstring tendons (Figure 1C).

### MiR-146a expression in rat tendons and tenocytes

**Figure 2:**
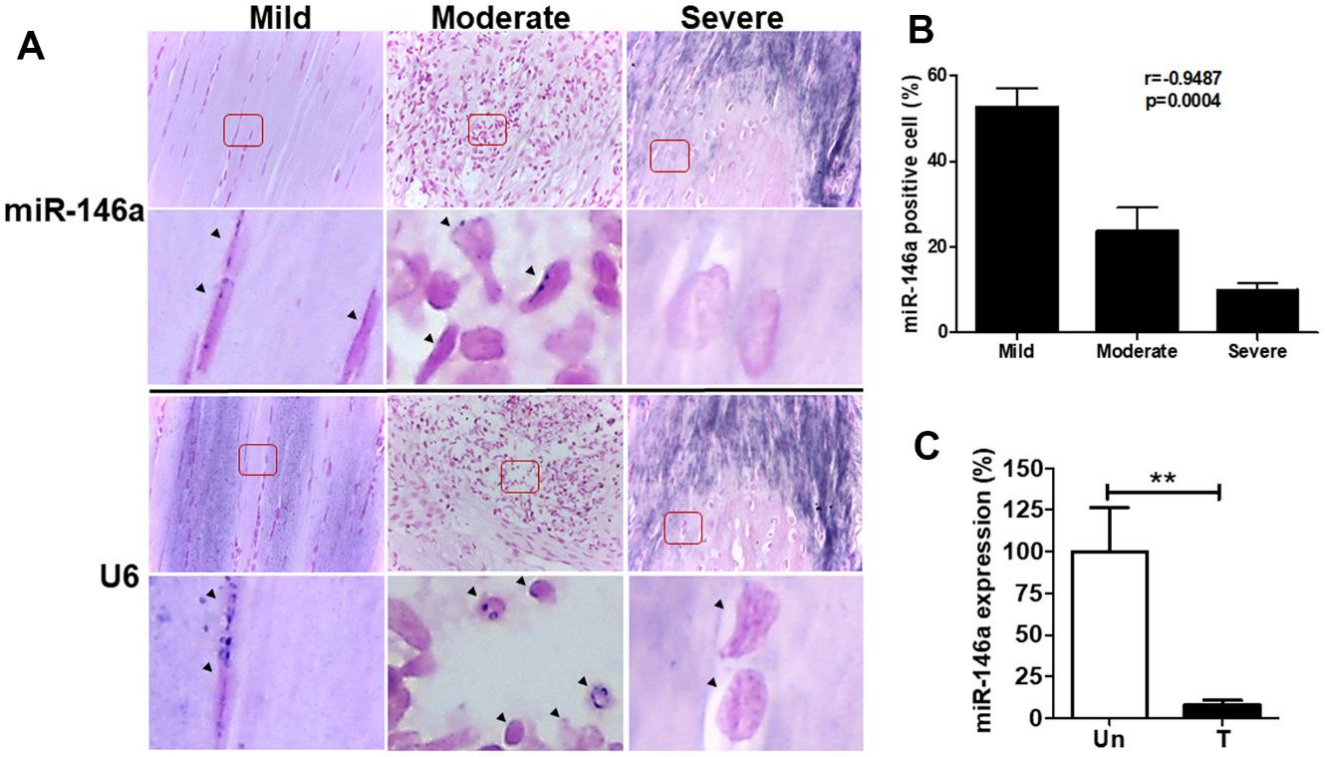
**miR-146a expression in tendon tissues and tenocytes from rats with tendinopathy.** Eight weeks after intratendinous injection of 10 μL collagenase I (0.015 mg/μL), the Achilles tendons were harvested from Sprague-Dawley rats. (**A**) Representative figures of *in situ* hybridization of miR-146a in rat tendinopathic tendons with different severity grades. Arrow heads indicate miR-146a- and U6-positive cells. Scale bars represent 50 μm in × 400 magnifications. The higher-magnification views of the red-boxed areas are shown beneath them. (**B**) miR-146a-positive cells were counted and normalized with U6-counterstained total cells (n=3). Spearman correlation rank test was used. (**C**) miR-146a expression levels in rat tendinopathic tenocytes primarily cultured from Achilles tendons treated (T) with collagenase I (0.015 mg/μL, 10 μL injection/rat) for 1 week or untreated (Un), as determined by qRT-PCR (n=3 for Un, n=5 for T). Values are represented as the mean ± SEM. **p<0.01.

We cultured primary rat tendinopathic tenocytes from Achilles tendons treated with collagenase for one week, following established protocols [3, 11]. Additionally, we utilized a rat collagenase-induced tendinopathy model based on previous studies [11, 23]. Similar to human tendons, rat miR-146a exhibited strong staining in tenocyte-like cells in the mild grade sections but was rarely observed in chondrocyte-like cells in the severe grade sections (Figure 2A). Quantitative analysis indicated a negative correlation between miR-146a expression levels and the histological grades of tendinopathy (r = -0.9487, p<0.001, Figure 2B). In our cell model, we isolated tenocytes from rat Achilles tendons and categorized them as treated (T) or untreated (Un) following intratendinous injection of collagenase [3, 11]. Treated tenocytes exhibited significantly lower miR-146a expression levels than untreated control tenocytes, as determined by qRT-PCR (Figure 2C).

### Lentiviral vector-mediated miR-146a gene transfer reduces senescence and SASPs in rat tendinopathic tenocytes

**Figure 3:**
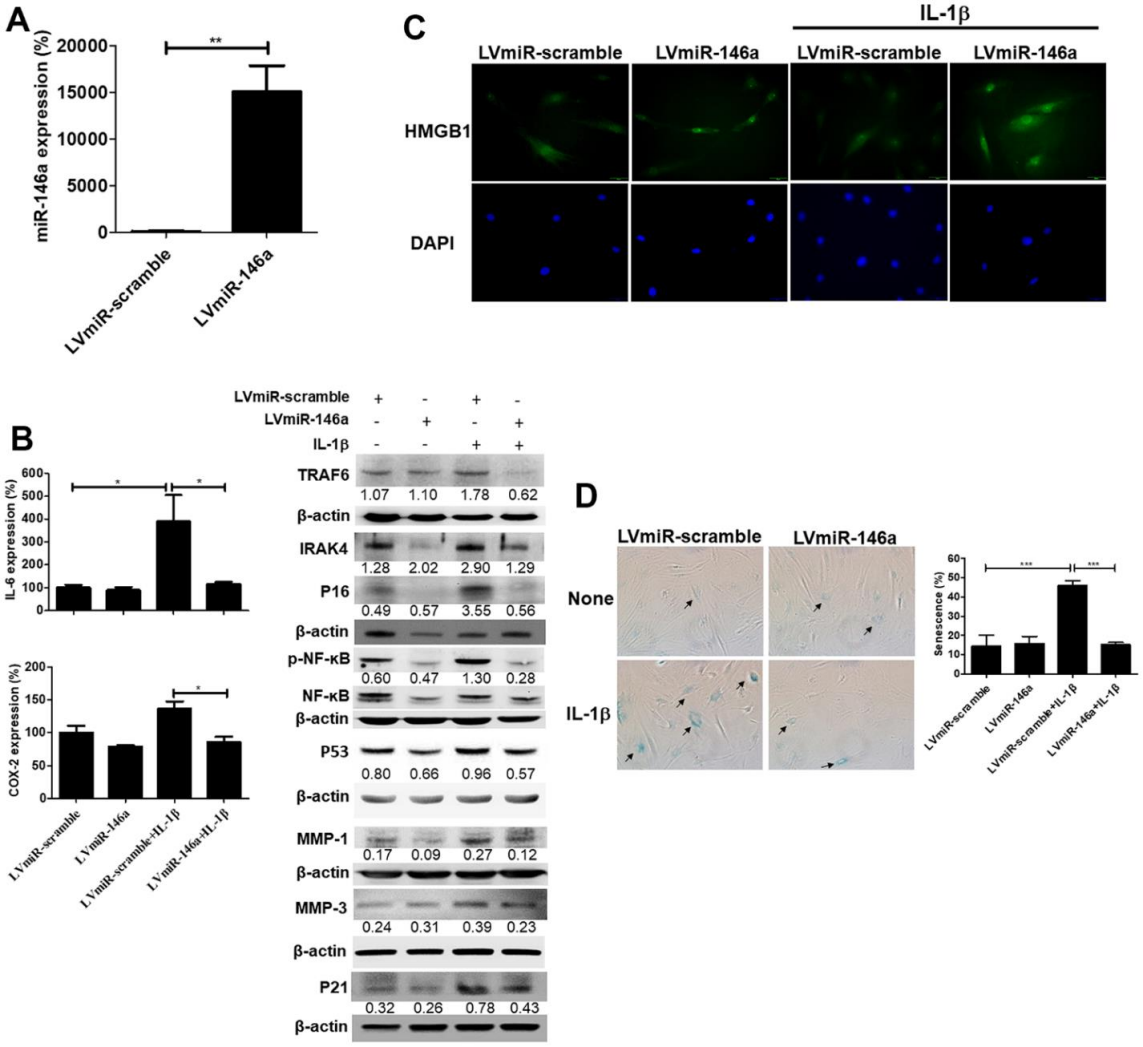
**Expression levels of miR-146a, senescent and senescence-associated secretory phenotype (SASP) markers, and senescence-associated β-galactosidase (SA-β-gal) activity in LVmiR-146a-transduiced rat primary tenocytes.** (**A**) QRT-PCR for miR-146a expression levels in LVmiR-scramble-(the control vector) and LVmiR-146a-infected rat tendinopathic tenocytes 72h post-infection. (**B**) LVmiR-146a- and LVmiR-scramble-transduced tenocytes were stimulated with IL-1β (10 ng/mL) or left unstimulated for 96 h. QRT-PCR for determining IL-6 and COX-2 expression levels. LVmiR-146a- and LVmiR-scramble-transduced tenocytes were stimulated with IL-1β (10 ng/mL) or left unstimulated for 24 h (n = 3). Immunoblotting for TRAF6, IRAK-4, phospho-NF-kB, NF-kB, p53, p21, p16, matrix metalloproteinase-1 (MMP)-1 and -3 expression levels in LVmiR-146a- and LVmiR-scramble-transduced tenocytes. (**C**) Representative figures of immunofluorescence staining for high mobility group box-1 (HMGB1) expression. LVmiR-146a- and LVmiR-scramble-transduced tenocytes were stimulated with IL-1β (10 ng/mL) or left unstimulated for 96 h. DAPI indicates nuclear staining. Scale bars shown at ×40 magnification correspond to 20 μm. (**D**) The tenocytes were subjected to subjected to SA-β-gal activity assay. SA-β-gal was identified and counted in five high-power fields (200×) to determine the average percentages of SA-β-gal-positive cells corresponding to total cells (n = 4). Results are representative of at least two independent experiments. Values are represented as the mean ± SEM. *p<0.05, ***p<0.001.

Rat tendinopathic tenocytes were transduced with lentiviral vectors expressing precursor miR-146a (LVmiR-146a) to investigate the effects on senescence and SASPs. Tenocytes with overexpressed miR-146a showed nearly 150-fold higher miR-146a levels than control transfectants (LVmiR-scramble), as determined by qRT-PCR (Figure 3A). Furthermore, LVmiR-146a-transduced tenocyte transfectants displayed decreased protein levels of p53, p21, p16, MMP-1, MMP-3, phospho-NF-κB/NF-κB ratio, TRAF6 and IRAK-4 compared to control transfectants (LVmiR-scramble) under IL-1β-stimulated conditions, as determined by immunoblotting (Figure 3B, right). Additionally, RNA levels of IL-6, cyclooxygenase-2 (COX-2) were lower in LVmiR-146a-transduced tenocyte transfectants than in their LVmiR-scramble-transduced counterparts in response to IL-1β stimulation (Figure 3B, left). Immunofluorescence showed a significantly higher nuclear staining ratio of high mobility group box 1 (HMGB1) in LVmiR-146a-infected tenocytes than in LVmiR-scramble-infected control cells in response to IL-1β stimulation (Figure 3C). Furthermore, these transfectants exhibited reduced Senescence β-Galactosidase (SA-β-gal) activity in response to IL-1β stimulation (Figure 3D).

## DISCUSSION

In this study, we demonstrated for the first time that miR-146a expression was negatively correlated with the severity of tendinopathy in human and rat tendons (Figures 1, 2). Over-expression of miR-146a by lentivirus-mediated gene transfer ameliorates senescence and SASPs in IL-1β stimulated tendinopathic tenocytes through reducing the levels of IRAK-4 and TRAF6, as well as enhancing nuclear localization of HMGB1. Furthermore, expression level of the inflammatory mediator, phospho-NF-ĸB was also decreased in the present experimental settings (Figure 3).

Emerging evidence highlights miRNAs as key regulators in the pathogenesis of tendinopathy. IL-33 induces the transition of collagen type 1 to 3 through NF-κB activation and down-regulation of miR-29a in human tenocytes [26]. Over-expression of miR-29a decreased the expression of type 3 collagen and improved early tendon injury in an equine model [27]. Additionally, treatment of miR-210 accelerated the healing of Achilles tendon by enhancing angiogenesis in a rat tendon injury model [28]. One study also indicated that miR-29b prevented the chitosan-induced tendon adhesion by regulating the TGF-β1 signaling pathway in a rodent Achilles tendon injury model [29]. These studies suggest that extracellular stimuli-mediated signaling pathways may exert their beneficial or pathologic effects on tendinopathy by regulating endogenous miRNA expression.

In particular, miR-146a was abundantly expressed in articular cartilage with a low Mankin score. Decreased expression of miR-146a correlated with decreasing COL2A1 and increasing MMP-13 expression patterns [30], and its induction by IL-1β occurred in a NF-κB-dependent manner in primary chondrocytes [31]. Interestingly, a study in human senescent fibroblasts suggested a feedback inhibition loop involving miR-146a/b and IL-1/NF-κB signaling, with miR-146a/b expression suppressing IL-6, and IL-8 secretion, as well as down-regulating IRAK-1 [32]. We found similar expression patterns in our study that miR-146a was highly expressed in tenocyte-like cells from the mild section, whereas it was rarely stained in chondrocyte-like cells from the severe section in both human and rat tendinopathic tendons (Figures 1, 2). Over-expression of miR-146a blocked the activation of NF-ĸB and decreased TRAF6 and IRAK-4 expression levels, thereby ameliorating senescence and SASPs in IL-1β -stimulated tenocytes (Figure 3). We have observed that IL-1β stimulates miR-146a expression in tendinopathic tenocytes (data not shown). These observations imply the existence of a feedback inhibition loop involving miR-146a and IL-1β/NF-κB signaling in senescent tenocytes, which requires further investigation.

HMGB1 belongs to the alarmin family and functions intracellularly However, upon cellular stress or damage, it is secreted by senescent cells, which occurs very early after a senescence-inducing stimulus, before development of SASPs [33, 34]. Extracellular HMGB1 can bind cell surface receptor for advanced glycation end products (RAGE) to initiate signaling cascades that promote the expression of IL-6 [35]. Furthermore, HMGB1 acquires proinflammatory potential by binding to IL-1β [36]. We showed that IL-1β-stimulated nuclear loss of HMGB1 could be compensated by over-expression of miR-146a in tendinopathic tenocytes (Figure 3C). IL-6 expression was reduced in miR-146a-transduced tendinopathic tenocytes upon IL-1β stimulation (Figure 3B). Therefore, we suggested that HMGB1 could be a potential alarmin for tenocyte senescence in tendinopathy.

Our previous study demonstrates that CD44 expression is positively correlated with the disease severity of human LHB tendinopathy, and over-expression of CD44 by lentiviral vectors ameliorates disease characteristics in a rat collagenase-induced Achilles tendinopathy model [3, 11]. We propose compensative and anti-senescent roles of CD44 in tendinopathy. HA/CD44 promotes cancer cell survival and motility via AKT activation [37, 38], which is in line with our previous findings showing decreased expression of phosphorylated AKT with poor motility in tendinopathic tenocytes by blocking CD44 with an antagonizing antibody [3]. MiR-146a activates AKT and subsequently leads to β-catenin stabilization in oral cancer stem cells [39]. Interestingly, the promoter regions of CD44 and miR-146a contain binding sites for β-catenin [39, 40], indicating that the two molecules can be trans-activated by the nuclear localization of β-catenin. We suggest positive feedback loops may exit in the AKT-β-catenin-CD44 and AKT-β-catenin-miR-146a axes. Taken together, the regulation between CD44 and miR-146a is worth exploring in tendinopathy, and AKT might be a critical signaling regulator between CD44 and miR-146a.

This study has some limitations. First, the mechanism involving the down-regulation of miR-146a expression during disease progression remains unclear (Figures 1, 2). A loss-of-function study using miR-146a sponge or antagomir can be designed in IL-1β-stimulated tenocytes to provide counterproof. Second, the *in vivo* effects of miR-146a should be evaluated. Our *in vitro* analysis revealed a protective effect of miR-146a against IL-1β-induced tenocyte senescence and SASPs (Figure 3). We propose that miR-146a may have therapeutic effects on rats with collagenase-induced Achilles tendinopathy, which will be investigated later.

## CONCLUSIONS

**Figure 4:**
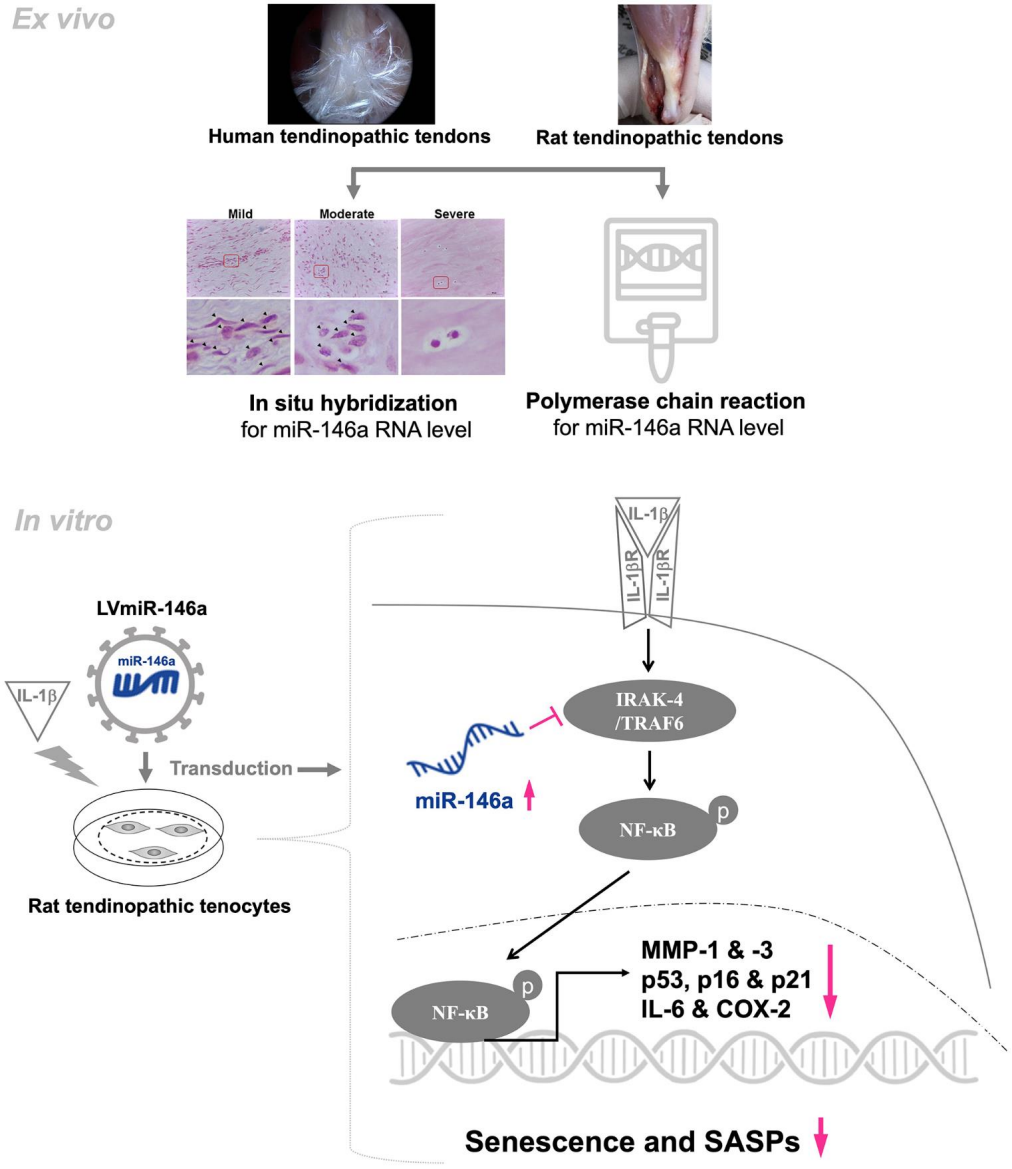
**Schematic overview of the working model for regulation of miR-146a in tendinopathy.** In human and rat tendinopathic tendons, miR-146a expression was examined by qRT-PCR and *in situ* hybridization. Compared with healthy controls, miR-146a expression was significantly lower. In tendinopathic tendons, miR-146a-positive cell ratio was negatively correlated with the severity of tendinopathy. Over-expression of miR-146a through lentivirus-mediated gene transduction (LVmiR-146a) ameliorates senescence and SASPs in IL-1β stimulated tendinopathic tenocytes by reducing the levels of p53, p16, p21, IL-6, MMP-1, and-3. The anti-inflammatory effect is achieved by lowering the levels of phospho-NF-ĸB and COX-2. Furthermore, in the present experimental settings, overexpression of miR-146a downregulates the expression levels of target molecules, including IRAK-4 and TRAF6.

In conclusion, we have demonstrated for the first time that miR-146a protects tendinopathic tenocytes from IL-1β-induced senescence and SASPs through the IRAK-4/TRAF6/NF-kB pathway (Figure 4). This study shed light on the role of miR-146a, which might prevent cell senescence in tendinopathy.

## Supplementary Material

Supplementary Table 1
