# Supplementary material for: MicroRNA-146a gene transfer ameliorates senescence and senescence-associated secretory phenotypes in tendinopathic tenocytes
Source: Aging (Albany NY). 2024 Feb 2;16(3):2702–14. doi: 10.18632/aging.205505 (PMC10911367; doi:10.18632/aging.205505)
Supplement: Supplementary Table 1 [file aging-16-205505-s001.pdf]

SUPPLEMENTARY TABLE

Supplementary Table 1. Demographic and histopathological features in enrolled patients and collected long head of biceps tendons.

Patient No.	Age	Gender	Histopathological severity
1	59	Male	Mild, Moderate, Severe
2	52	Male	Mild, Moderate
3	69	Male	Moderate, Severe
4	71	Female	Mild, Moderate
5	63	Female	Severe
6	71	Female	Mild, Moderate, Severe
7	74	Female	Mild, Moderate, Severe
8	71	Female	Mild, Moderate, Severe
9	68	Female	Moderate, Severe*
10	63	Female	Moderate, Severe*

*No. 9 and No. 10 patients did not exhibit any miR-146a-positive cells in any histopathological severity field; hence, they were not included in the analysis of *in situ* hybridization for miR-146a. However, the miR-146a expression levels in these two patients could be detected in their specimens, and consequently, they were included in the analysis of miR-146a expression.
